# Supplementary material for: A fault-intrusion-tolerant system and deadline-aware algorithm for scheduling scientific workflow in the cloud
Source: PeerJ Comput Sci. 2021 Nov 2;7:e747. doi: 10.7717/peerj-cs.747 (PMC8576554; doi:10.7717/peerj-cs.747)
Supplement: Supplemental Information 6 [file peerj-cs-07-747-s006.zip › Myproject1.1/docs/org/workflowsim/clustering/balancing/methods/BalancingMethod.html]

BalancingMethod


JavaScript is disabled on your browser.


- Overview
- Package
- Class
- Use
- Tree
- Deprecated
- Index
- Help

- Prev Class
- Next Class

- Frames
- No Frames

- All Classes

- Summary:
- Nested |
- Field |
- Constr |
- Method

- Detail:
- Field |
- Constr |
- Method


org.workflowsim.clustering.balancing.methods

## Class BalancingMethod

- java.lang.Object
- - org.workflowsim.clustering.balancing.methods.BalancingMethod

- Direct Known Subclasses:
  :   ChildAwareHorizontalClustering, HorizontalImpactBalancing, HorizontalRandomClustering, HorizontalRuntimeBalancing, VerticalBalancing

  ---

    

  ```
  public class BalancingMethod
  extends java.lang.Object
  ```

  BlancingMethod is the super class of all balancing methods

  Since:
  :   WorkflowSim Toolkit 1.0

- - ### Constructor Summary

    Constructors

    Constructor and Description

    `BalancingMethod(java.util.Map levelMap,
    java.util.Map taskMap,
    int clusterNum)`

    Initialize a BalancingMethod
  - ### Method Summary

    Methods

    | Modifier and Type | Method and Description |
    | --- | --- |
    | `void` | `addTaskSet2TaskSet(TaskSet tail, TaskSet head)` Add all the tasks in tail to head and then clean tail. |
    | `void` | `cleanTaskSetChecked()` Clean all the check points |
    | `int` | `getClusterNum()` Gets clusters.num |
    | `java.util.Map` | `getLevelMap()` Gets the level map |
    | `java.util.Map` | `getTaskMap()` Gets the task map |
    | `void` | `run()` BalancingMethod is the super class and it should not be used. |

    - ### Methods inherited from class java.lang.Object

      `clone, equals, finalize, getClass, hashCode, notify, notifyAll, toString, wait, wait, wait`

- - ### Constructor Detail


    - #### BalancingMethod

      ```
      public BalancingMethod(java.util.Map levelMap,
                     java.util.Map taskMap,
                     int clusterNum)
      ```

      Initialize a BalancingMethod

      Parameters:
      :   `levelMap,` - taskSets per level
      :   `taskMap,` - map from task to taskSet
      :   `clusterNum` - number of clustered jobs
  - ### Method Detail


    - #### getTaskMap

      ```
      public java.util.Map getTaskMap()
      ```

      Gets the task map

      Returns:
      :   task map


    - #### getLevelMap

      ```
      public java.util.Map getLevelMap()
      ```

      Gets the level map

      Returns:
      :   level map


    - #### getClusterNum

      ```
      public int getClusterNum()
      ```

      Gets clusters.num

      Returns:
      :   clusters.num


    - #### addTaskSet2TaskSet

      ```
      public void addTaskSet2TaskSet(TaskSet tail,
                            TaskSet head)
      ```

      Add all the tasks in tail to head and then clean tail. Can be reused with
      verticalClustering()

      Parameters:
      :   `tail` -
      :   `head` -


    - #### run

      ```
      public void run()
      ```

      BalancingMethod is the super class and it should not be used.


    - #### cleanTaskSetChecked

      ```
      public void cleanTaskSetChecked()
      ```

      Clean all the check points


- Overview
- Package
- Class
- Use
- Tree
- Deprecated
- Index
- Help

- Prev Class
- Next Class

- Frames
- No Frames

- All Classes

- Summary:
- Nested |
- Field |
- Constr |
- Method

- Detail:
- Field |
- Constr |
- Method
